# Intraoperative imaging of a combined minimally invasive therapy for aortic valve stenosis with hypertrophic obstructive cardiomyopathy: a case report

**DOI:** 10.1007/s12928-025-01194-7

**Published:** 2025-09-26

**Authors:** Xiaoxue Zhang, Shiliang Li, Zijun Chen, Yi feng, Xiang Wei, Cai Cheng

**Affiliations:** 1 Tongji Hospital, Tongji Medical College, Huazhong University of Science and Technology, Wuhan, 430030 Hubei China; 2 Division of Cardiothoracic and Vascular Surgery, The Second Affiliated Hospital, Zhejiang University School of Medicine, Hangzhou, 310000 Zhejiang China; 3 Wuhan Taikang Tongji Hospital, Wuhan, 430050 Hubei China

A 65-year-old woman presented with dizziness and a history of stroke. Echocardiography, CT, and MRI revealed severe aortic valve stenosis (AS) with a peak instantaneous transvalvular gradient of 111 mmHg on TEE (Fig. 1A), and demonstrated aortic annular cross-sectional areas of 403.8 and 386.5 mm^2^ in systole and diastole, respectively (Fig. 1B). Further evaluation identified hypertrophic obstructive cardiomyopathy (HOCM), with a septal thickness of 19 mm and a left ventricular outflow tract (LVOT) gradient of 61 mmHg (Fig. 1C). CT angiography confirmed a mean systolic annular diameter of 23.0 mm (Fig. 1B).

**Fig. 1:**
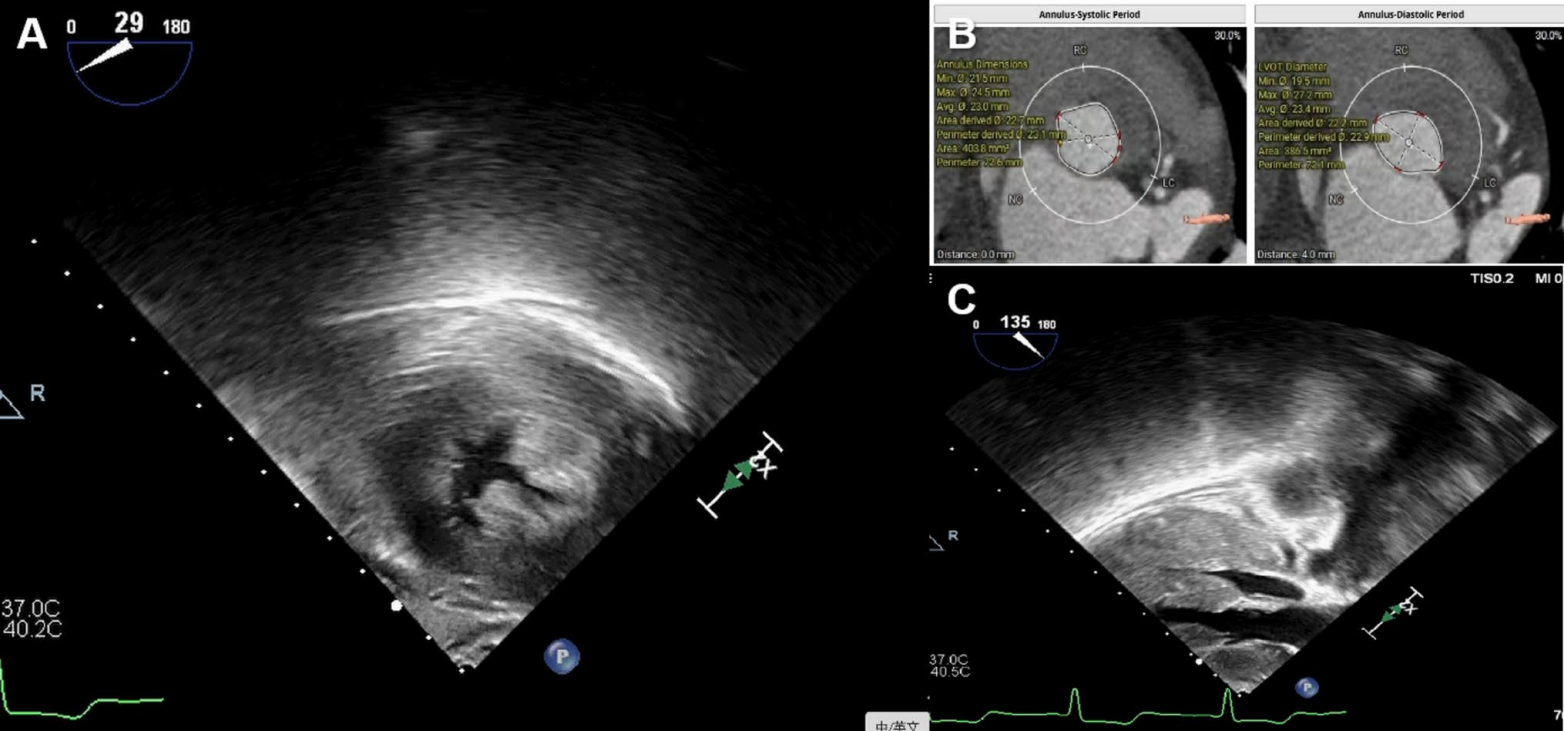
Transesophageal echocardiographic (TEE) and computed tomography (CT) images demonstrating aortic valve stenosis and hypertrophic obstructive cardiomyopathy (HOCM). **A** Shows restricted aortic valve opening with thickened cusps and dynamic left ventricular outflow tract obstruction due to asymmetric septal hypertrophy on TEE. **B** Presents CT-based measurements of the aortic annulus during diastole and systole, including annular diameters and areas, aiding in procedural planning. **C** Demonstrates the left ventricular outflow tract is narrowed due to asymmetric septal hypertrophy, consistent with dynamic obstruction

Perioperative risk (EuroSCORE II 7.99%) was driven primarily by COPD, residual post-stroke frailty with limited mobility, and insulin-dependent diabetes; the patient was NYHA class III with LVEF 55%.

With advances in less-invasive structural interventions, transapical beating-heart septal myectomy (TA-BSM, details in Fig. 2) [1] has emerged as a complementary option for patients with concomitant HOCM and AS undergoing transcatheter aortic valve implantation (TAVI). The beating-heart myectomy device (BMD, Wei-Xin-Tan Corp, China) is manufactured in models defined by the resection-window length and the sleeve-tube diameter (length/diameter, mm): 11/30, 11/40, 13/30, and 13/40. In this case, an 11/30-mm device was used. In the published first-in-human series [1], the extent of septal resection was reported by weight (not by area), with a median resected myocardial weight of 4.1 g (IQR 2.5–6.0 g; range 1.0–17.9 g). The resected myocardial weight in our case was 3.9 g.

**Fig. 2:**
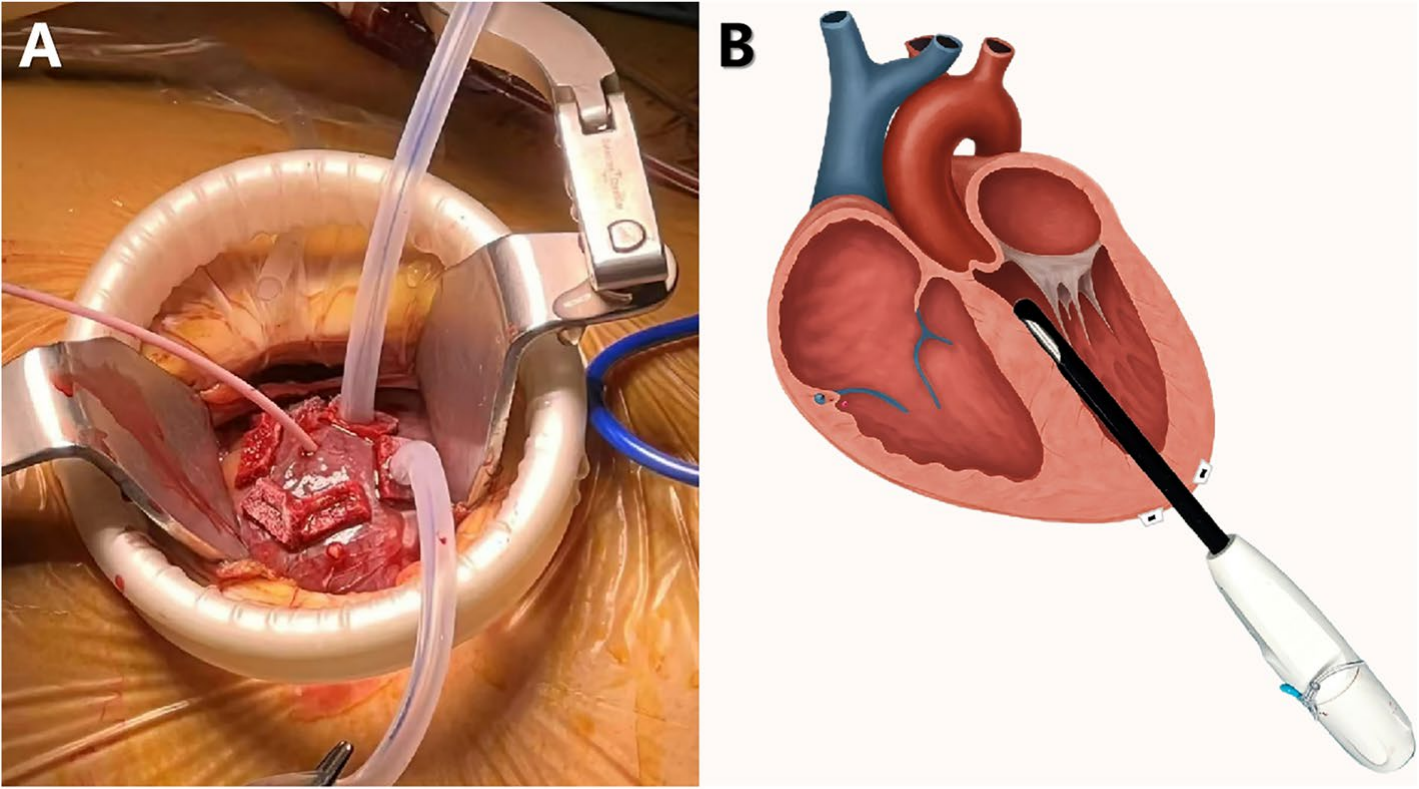
The beating-heart myectomy device (BMD). An apical puncture was produced inside the purse string and was dilated with the use of a dilator along a guidewire (**A**). After de-airing, the BMD in the off-state was introduced into the LVOT through the apical puncture under the navigation of TEE (**B**)

After induction of general anesthesia and left anterolateral mini-thoracotomy, two concentric apical purse-strings are placed; the apex is punctured and dilated to introduce the BMD. Real-time TEE guides a closed view → measurement → decision loop:

**Localization/trajectory:** Mid-esophageal long-axis (~ 110–130°) aligns the LVOT–AV–IVS axis and confirms a 5–10 mm safety margin below the aortic annulus; transgastric basal short axis refines the BMD cutting-window orientation and apposition to the most hypertrophied septum.

**First resection + reassessment:** Initial myectomy in the basal anterior septum, centered 5–10 mm sub-annular; immediate TEE reassessment documents LVOT peak instantaneous/mean gradients, SAM, MR, and septal thickness.

**Sequential passes:** Second pass rotated ~ 60–120°clockwise and slightly more basal to create a shallow, parallel groove channel; if needed, extend toward the mid-septum under long-axis guidance with gentle apex-to-base traction toward the apex before further cuts.

**Dual endpoints:** Procedural target: LVOT gradient < 30 mmHg at rest and < 50 mmHg with provocation, with MR ≤ 1 + ; if isoproterenol or Valsalva reveals residual obstruction, perform targeted additional resection until thresholds are met.

**Safety boundaries:** Avoid over-resection near the basal posterior septum (conduction system) to reduce postoperative LBBB risk; maintain clear visualization away from papillary muscles/chordae, using a mid-transgastric short-axis view as needed.

After 3 resections, the LVOT gradient reduced from 61 to 7 mmHg, and the septal thickness decreased from 19 to 11 mm. The echocardiogram clearly showed the cross-section of the myocardial incision (Fig. 3B).

**Fig. 3:**
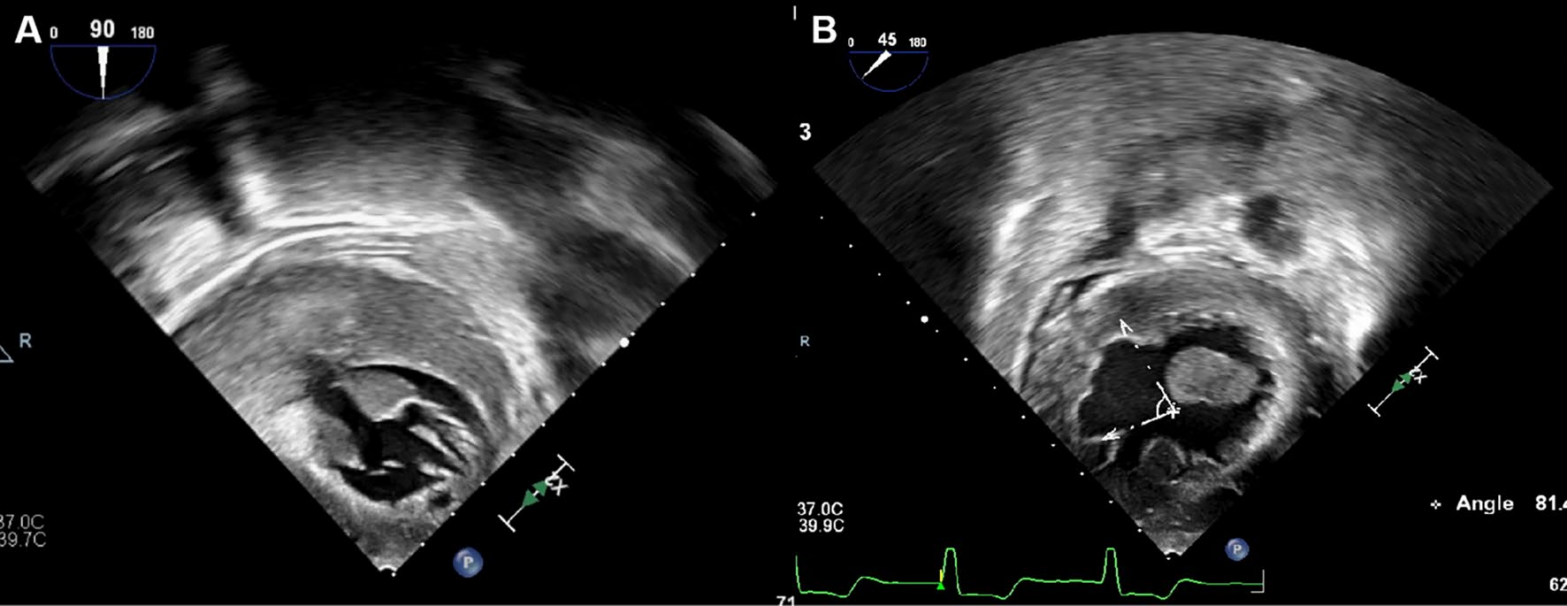
TEE showed asymmetric septal hypertrophy before TA-BSM (**A**). After TA-BSM, three resections was performed in the basal anterior septum (**B**)

Subsequently, the patient underwent TAVI. Under the guidance of DSA, we inserted a guide wire through the incision of the femoral artery to measure the aortic valve pressure, which was 64 mmHg. A balloon (18 × 40 mm) was delivered to predilate the stenotic valve. Sizing was based on the systolic CT annular diameter/area matched to the manufacturer’s sizing chart, and a 24-mm prosthesis (MicroPort, China; stent height 50 mm) was implanted. Post-procedural TEE showed a mean transvalvular gradient of 28 mmHg with trivial paravalvular regurgitation (Fig. 4B), while the catheter-derived mean gradient decreased to 0 mmHg. A pre-discharge ultrasound showed resolution of both stenosis and regurgitation. At 3-month follow-up, findings remained stable.

**Fig. 4:**
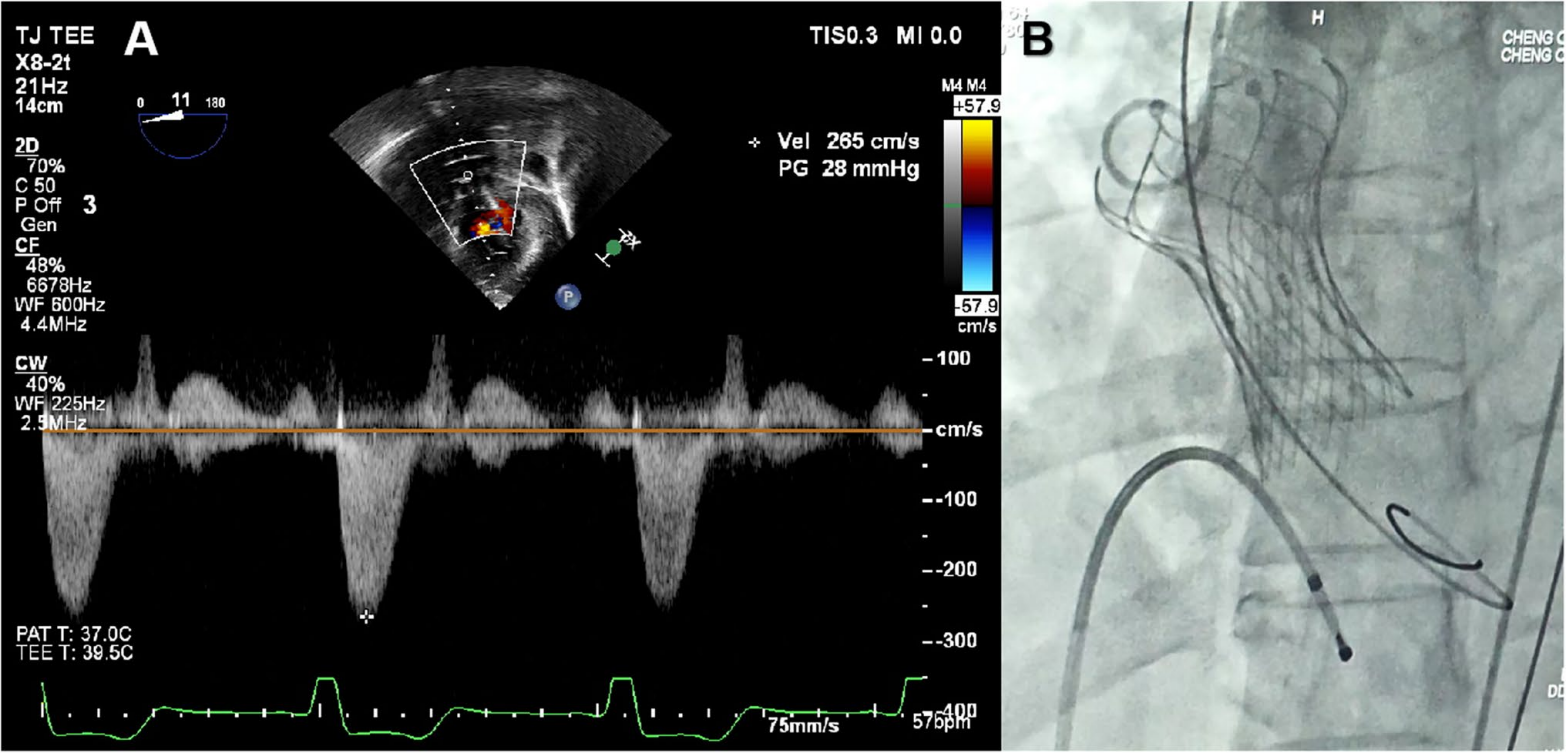
After TAVI, the transesophageal echocardiographic image demonstrated the successfully implanted transcatheter aortic valve with a mean transvalvular pressure gradient of 28 mmHg. The corresponding digital subtraction angiography (DSA) confirmed satisfactory valve deployment and preserved coronary perfusion

To our knowledge, this represents the first documented case of combined TA-BSM and TAVI in a patient with coexisting HOCM and severe AS. The successful outcome highlights the potential of this minimally invasive strategy for selected high-risk patients.

## Data Availability

The data that support the findings of this study are available from the corresponding author upon reasonable request.
